# Supplementary material for: Visualization of a multi-turnover Cas9 after product release
Source: Nat Commun. 2025 Jul 1;16:5681. doi: 10.1038/s41467-025-60668-7 (PMC12217974; doi:10.1038/s41467-025-60668-7)

Supplementary Information

Visualization of a multi-turnover Cas9 after product release

Kaitlyn A. Kiernan[1], David W. Taylor[1-4]

[1] Department of Molecular Biosciences, University of Texas at Austin, Austin, TX, USA
[2] Institute for Cellular and Molecular Biology, University of Texas at Austin, Austin, TX, USA
[3] Center for Systems and Synthetic Biology, University of Texas at Austin, Austin, TX, USA
[4] Livestrong Cancer Institutes, Dell Medical School, Austin, TX, USA

*Corresponding author. Email: dtaylor@utexas.edu

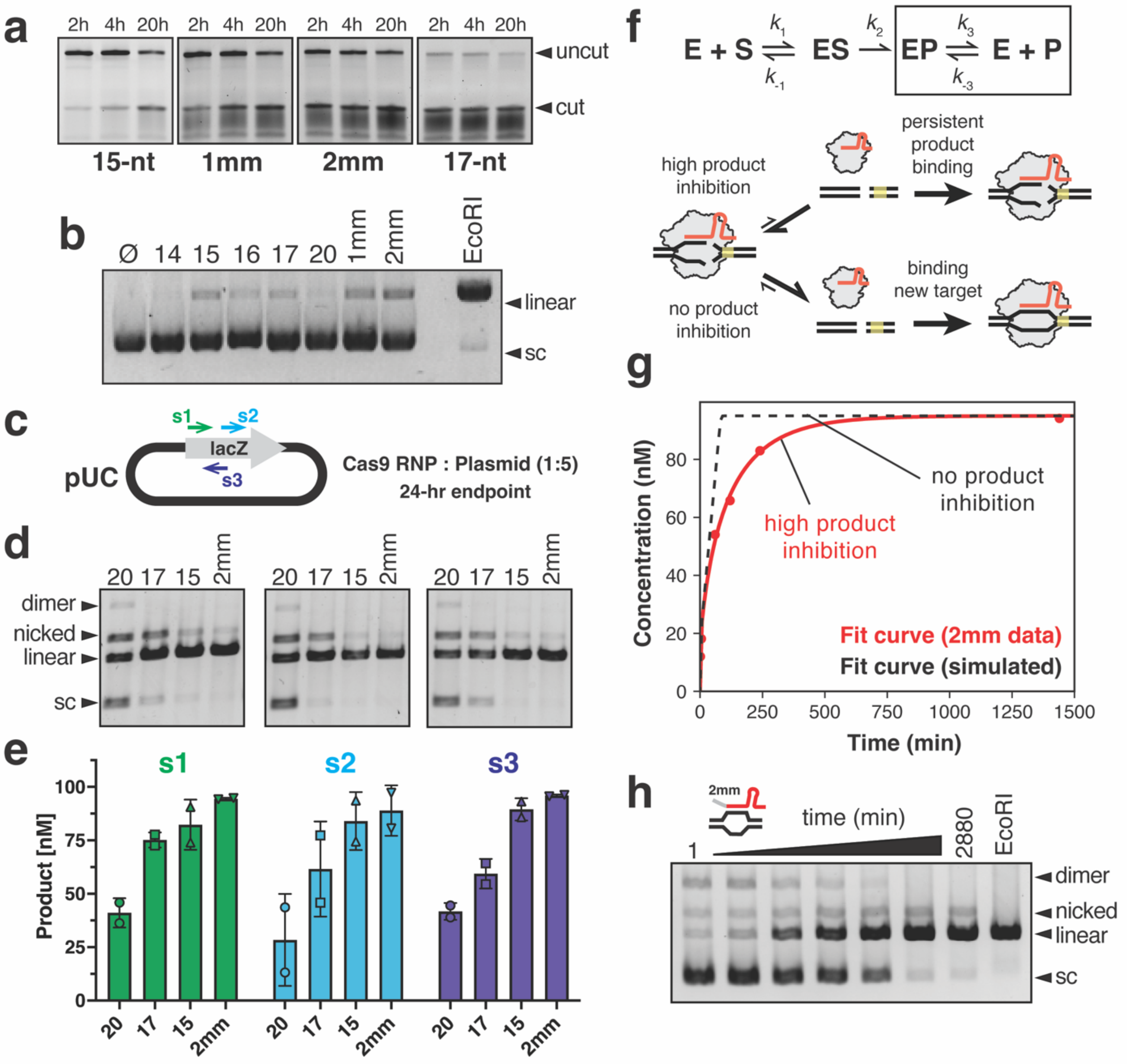

Supplementary Figure 1. Kinetic analysis of Cas9 programmed with truncated sgRNAs. (a) Cleavage of a fluorescently labeled 55-bp dsDNA target with a 15-nt, 2mm, 17-nt, and 20-nt sgRNA. Cas9 RNP was added in excess (50 nM) to the dsDNA target (10nM). Cleavage products were analyzed on 15% polyacrylamide denaturing gels. **(b)** Cas9 cleavage assay with plasmid DNA in 5X molar excess (20 nM RNP: 100 nM plasmid). Linear product measured following 18 hours of incubation with 14-, 15-, 16-, 17-, and 20-nt sgRNAs, and with a 16-nt sgRNA containing one mismatch (1mm) and 17-nt sgRNA with two terminal mismatches (2mm). **(c)** Schematic showing spacer sites along the plasmid substrate. Spacer 1 (s1) is the same spacer used in all other cleavage assays in this study. Spacer 2 and 3 (s2 and s3) target different sites in the lacZ gene on the pUC19 plasmid. **(d)** Cleavage of plasmids using s1, s2, and s3 where 20 nM active Cas9 RNP was added to 100 nM plasmid and product generation was measured at 24-h. **(e)** Quantification of total linear product from cleavage assays in panel d. Data represented

as the mean ± st. dev. with n = 2. **(f)** Equation used to fit kinetic data with box around the product release step of the reaction. Schematic below showing difference in reaction outcome with high degrees of product inhibition or no product inhibition present within the reaction. **(g)** Kinetic curves of experimental data (2mm) from the plasmid cleavage assays displaying high degrees of product inhibition overlayed with simulated data generated in Kintek Explorer showing what the curve would look like without any product inhibition. **(h)** Turnover assay with the 2mm sgRNA sample. 20 nM active Cas9 RNP was added to 100 nM plasmid substrate and product generation was measured at 1, 5, 60, 120, 1440, and 2880 minutes. Fully-digested plasmid with EcoRI used as a control. Cleavage products were visualized using 1% agarose gels post-stained with 3X GelRed.

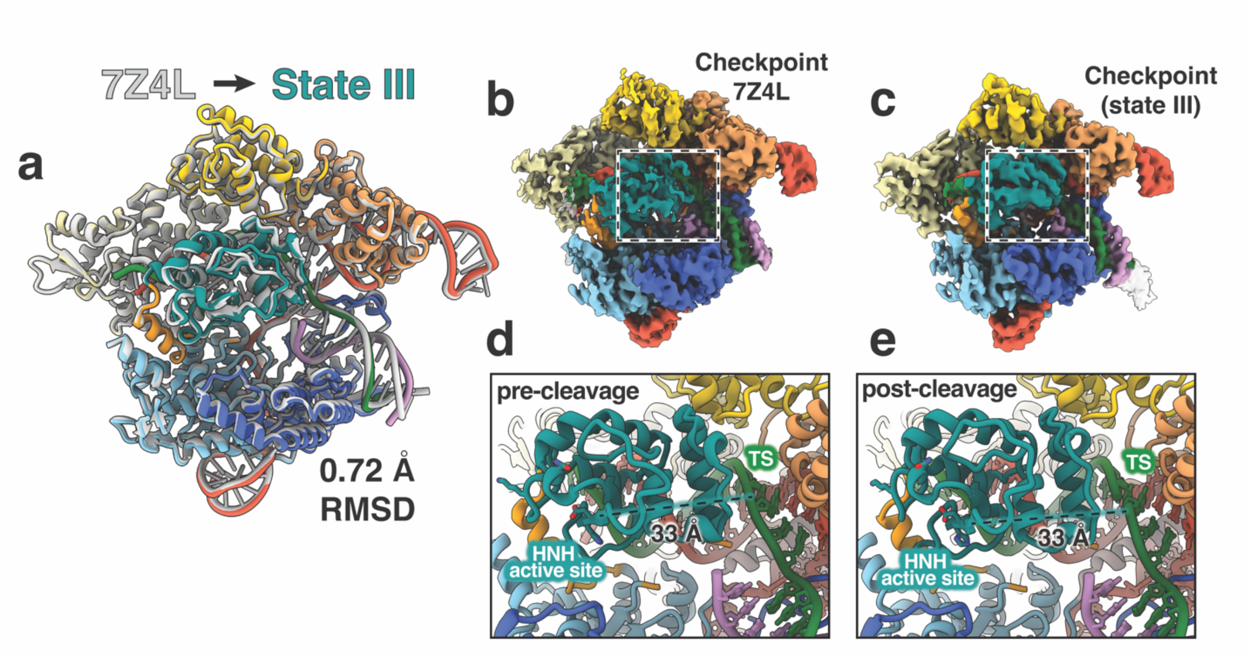

Supplementary Figure 2. Analysis of the conformational checkpoint state. (a) Overlay of PDB 7Z4L with the conformational checkpoint state (state III) in our dataset. RMSD calculated in ChimeraX across all equivalent C-alpha atoms. **(b-c)** Cryo-EM maps colored by domain of the checkpoint state from **(b)** Pacesa et al 2022 and **(c)** this study. **(d)** Checkpoint structure before docking onto the TS for cleavage. The active site residues shown as sticks and is ~ 33 Å away from the TS. **(e)** Checkpoint structure directly following DNA cleavage after HNH is undocked from the TS. HNH adopts the same conformation as seen before cleavage with the active site rotated away ~ 33 Å from the cleaved TS.

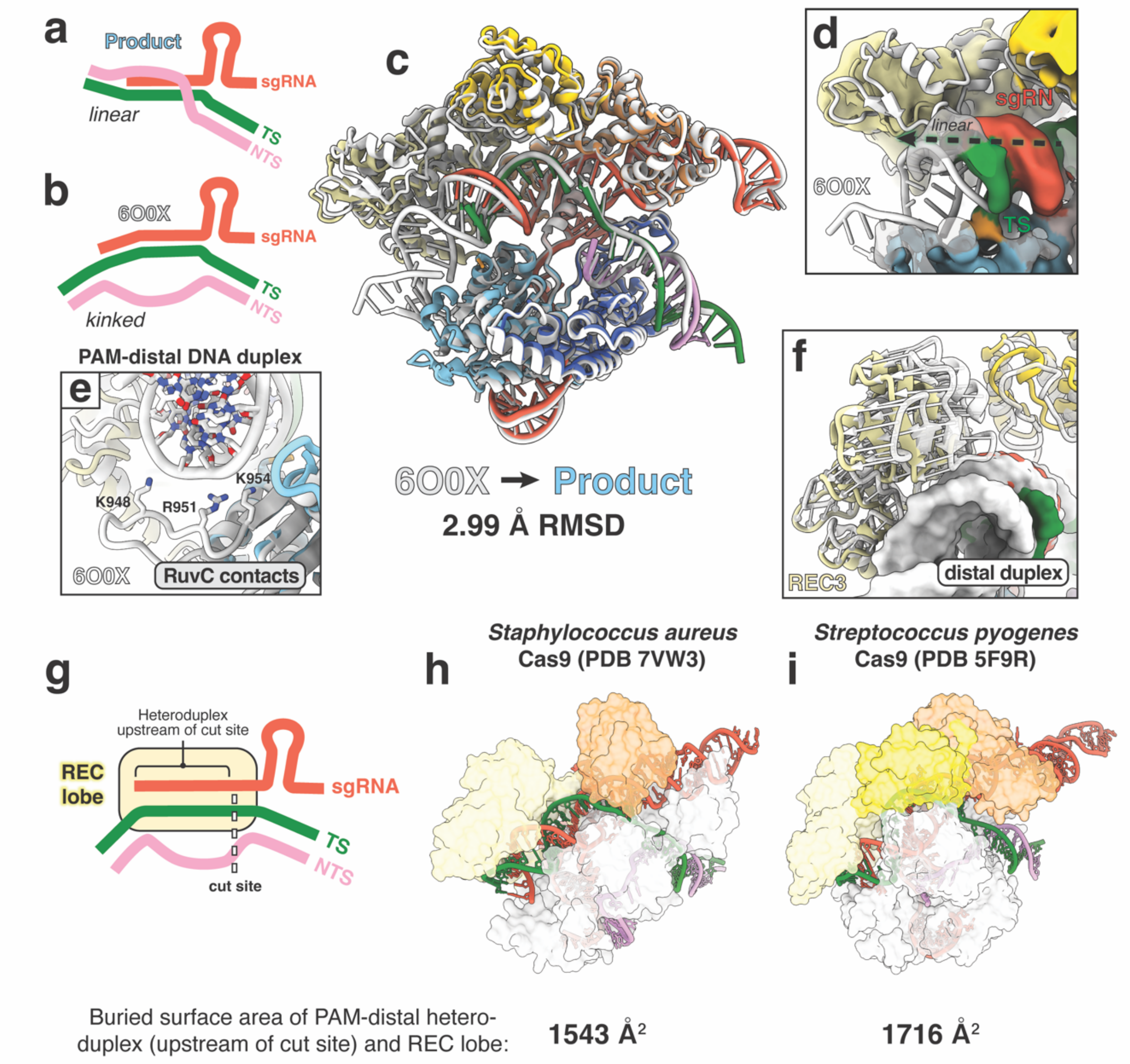

Supplementary Figure 3. Structural comparison of product state. (a) Schematic of the linear R-loop. **(b)** Schematic of the kinked R-loop. **(c)** Overlay of PDB 6O0X with the product state from our dataset. **(d)** Low-pass filtered product state map (6 Å) shows the path of the DNA duplex follows a linear trajectory compared with the kinked trajectory observed in 6O0X. **(e)** In 6O0X, electrostatic interactions in the RuvC domain are established with the PAM-distal DNA. These interactions are not observed in our product state structure. **(f)** Modevector arrows showing REC3 movement away from the heteroduplex in our product state (yellow) whereas in the 6O0X structure, REC3 remains docked onto the PAM-distal heteroduplex (white). (**g**) Schematic of the REC lobe footprint along the heteroduplex. Dashed line indicates cut site. **(h)** *Staphylococcus aureus* Cas9 (PDB 7VW3) shown as transparent surface with the REC lobe colored by domain. Buried surface area (SA) between the heteroduplex upstream of the cut site and the REC lobe (residues 74-447) shown below. **(i)** *Streptococcus pyogenes* Cas9 (PDB 5F9R) shown as transparent surface with the REC lobe colored by domain. Buried SA calculated between the heteroduplex upstream of the cut site and REC lobe (residues 95-731) shown below.

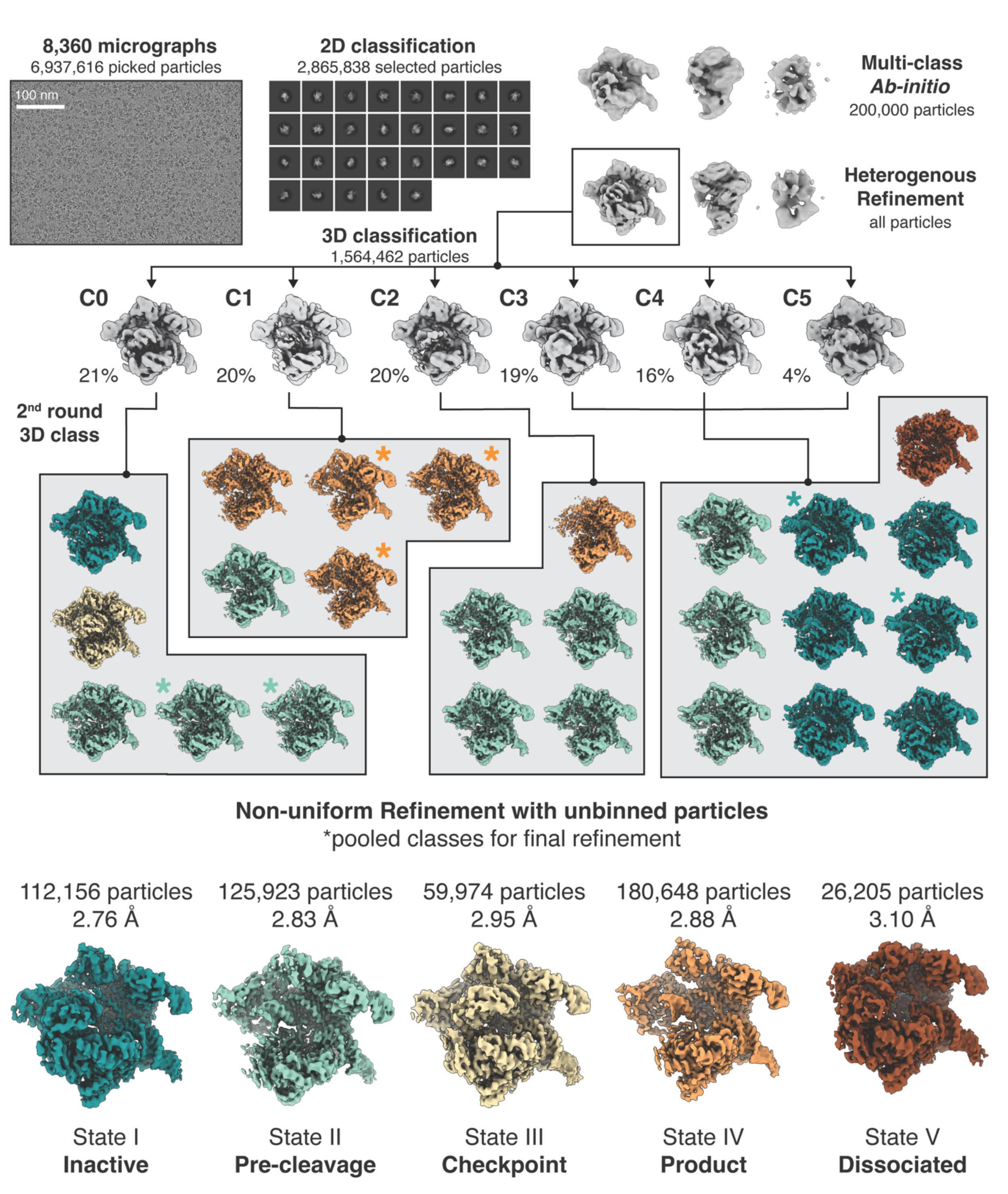

Supplementary Figure 4. Cryo-EM data analysis workflow.

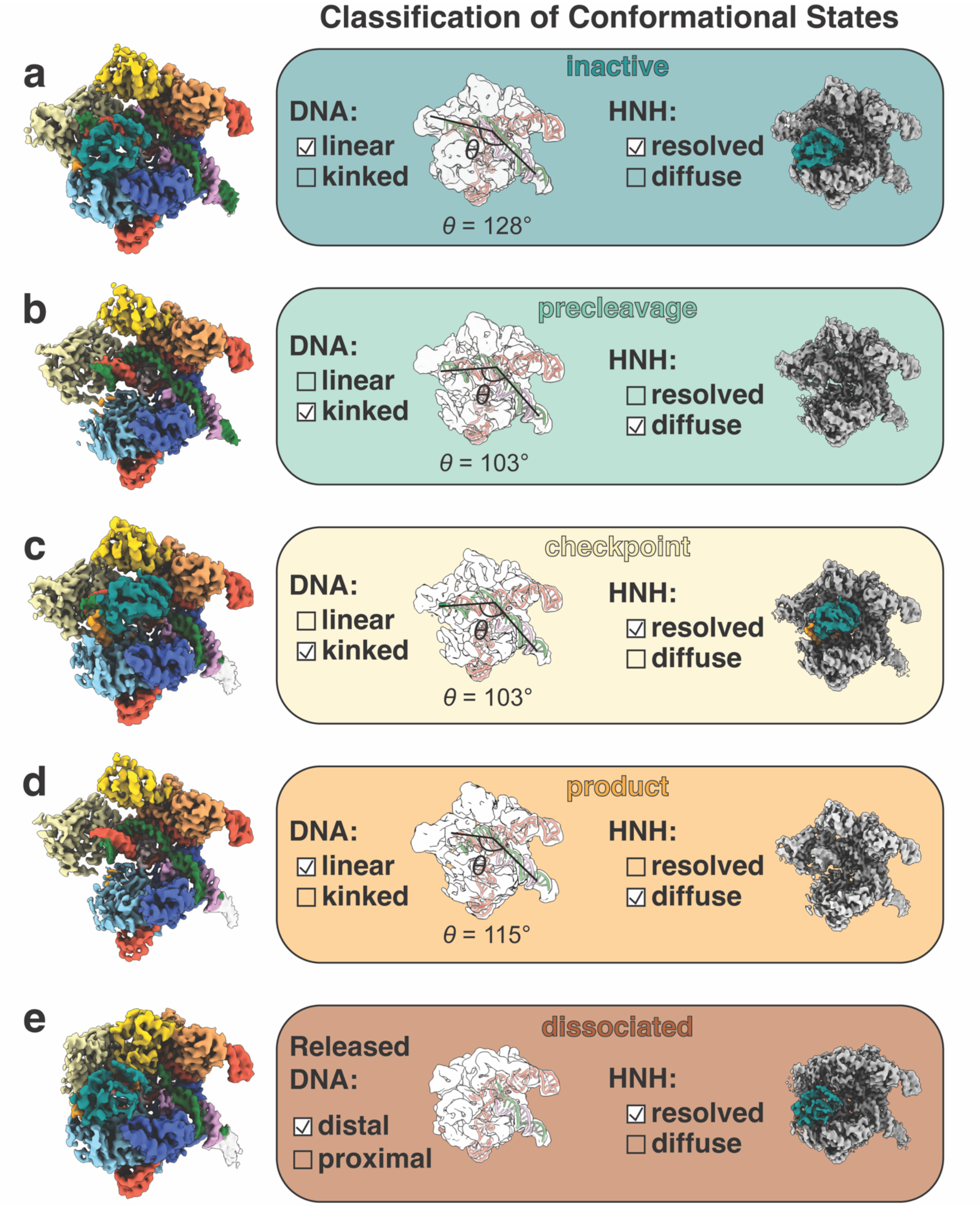

Supplementary Figure 5. Conformational state assignment criteria. (a-e) Metrics used to manually inspect and classify subclasses into the inactive, pre-cleavage, checkpoint, product and dissociated states. **(a)** In the inactive state, the DNA duplex adopts a linear conformation and the HNH domain is clearly resolved and remains docked

onto the NUC lobe. **(b)** In the pre-cleavage state, the DNA adopts a kinked conformation and triggers undocking of the HNH domain. HNH is flexible as it reorganizes and is not resolved. The TS remains intact. **(c)** In the checkpoint conformation, the DNA is still kinked but the TS has been cleaved. The HNH domain is clearly resolved and has undocked from the cut TS. **(d)** In the product state, the DNA has been cleaved and the PAM-distal duplex adopts an intermediate conformation in-between the linear and kinked states observed in the inactive and pre-cleavage states. HNH is flexible and is not resolved in this state. **(e)** In the dissociated state, the PAM-distal DNA is released and the HNH domain is clearly observed to be reset into the position as observed in the binary complex.

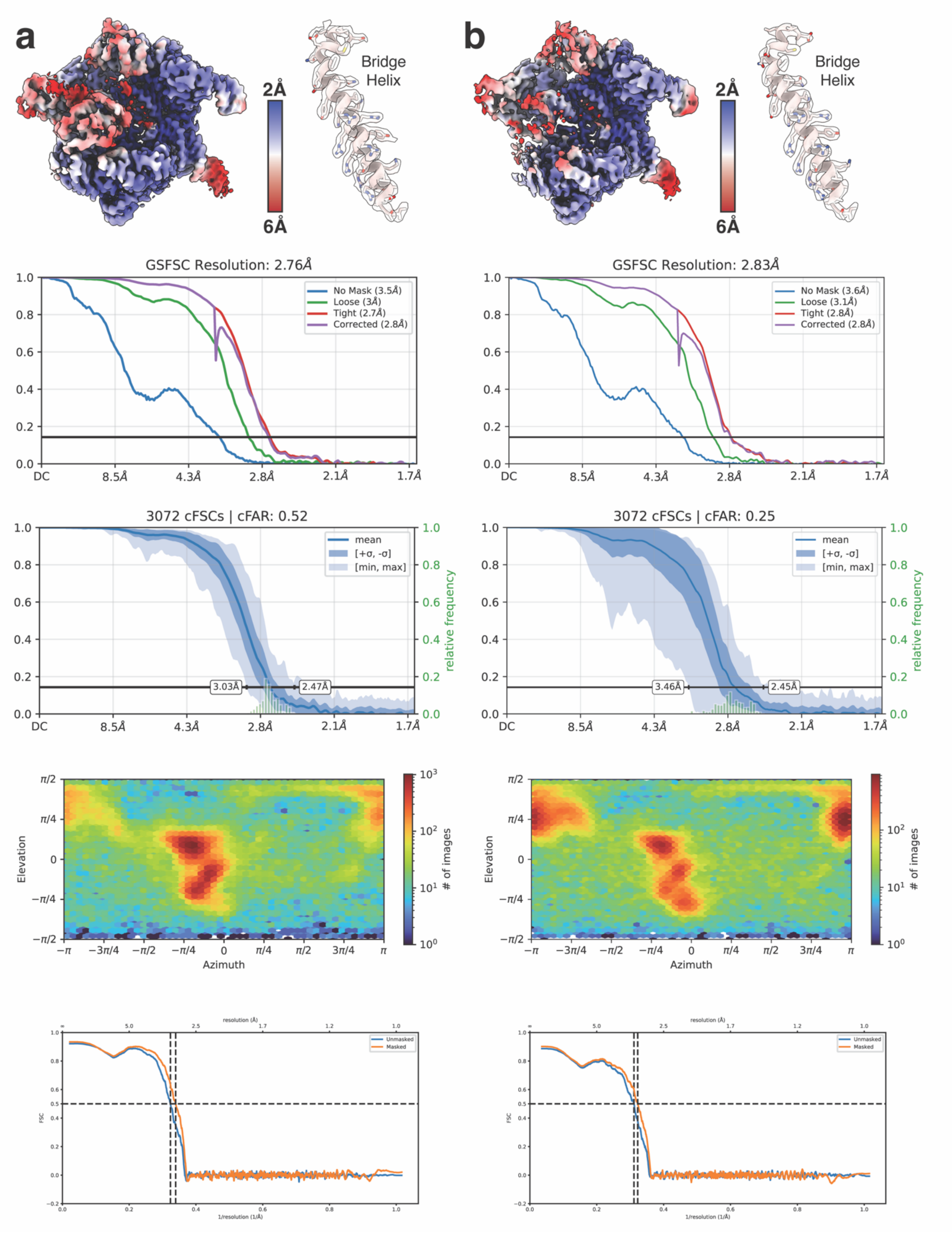
a
b
Bridge Helix
2Å
6Å
GSFSC Resolution: 2.76Å
GSFSC Resolution: 2.83Å
No Mask (3.5Å)
Loose (3Å)
Tight (2.7Å)
Corrected (2.8Å)
No Mask (3.6Å)
Loose (3.1Å)
Tight (2.8Å)
Corrected (2.8Å)
3072 cFSCs | cFAR: 0.52
3072 cFSCs | cFAR: 0.25
mean
[+σ, -σ]
[min, max]
relative frequency
3.03Å
2.47Å
3.46Å
2.45Å
Elevation
Azimuth
# of images
Unmasked
Masked
resolution (Å)
1/resolution (1/Å)
FSC

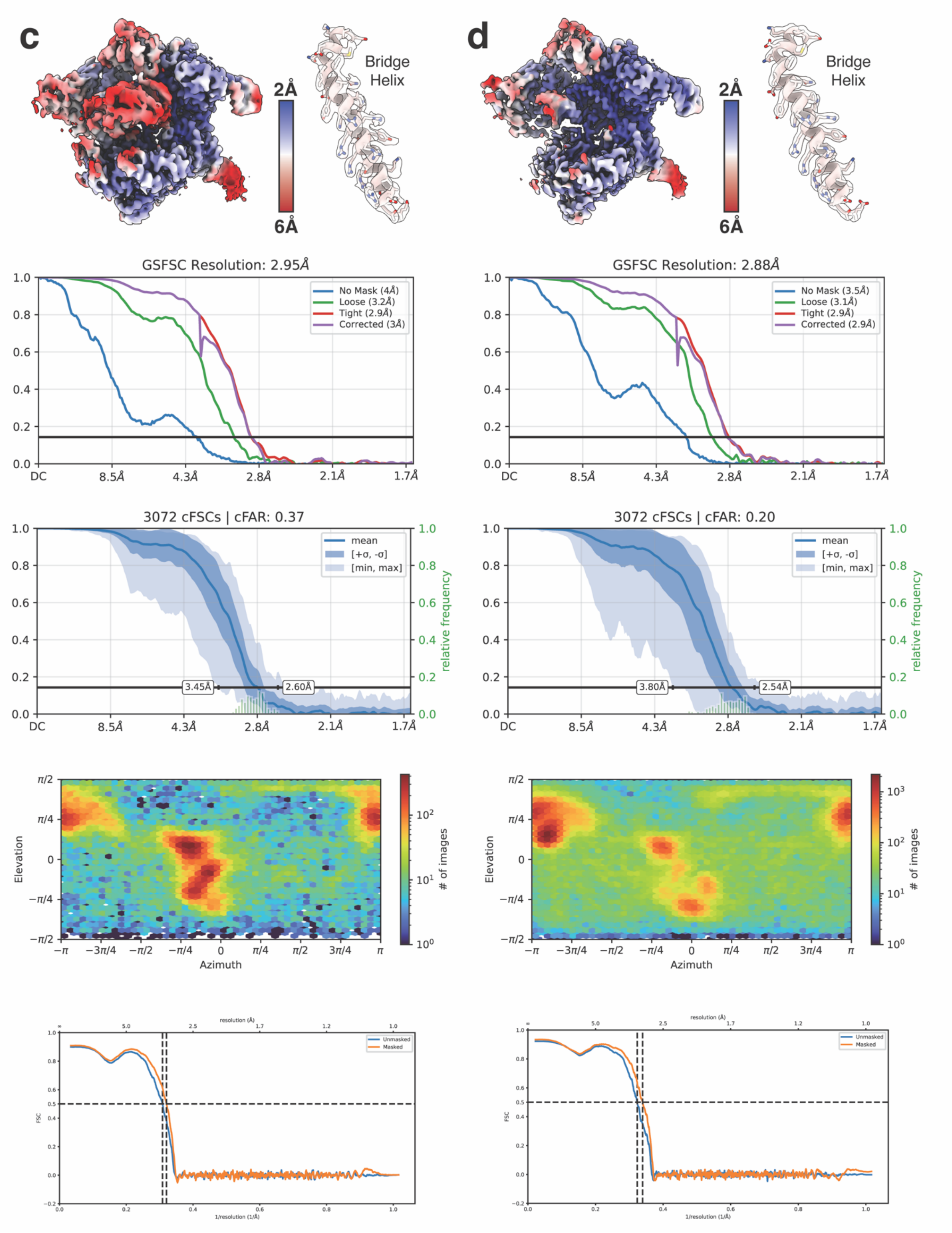
c
Bridge Helix
2Å
6Å
GSFSC Resolution: 2.95Å
No Mask (4Å)
Loose (3.2Å)
Tight (2.9Å)
Corrected (3Å)
DC
8.5Å
4.3Å
2.8Å
2.1Å
1.7Å
3072 cFSCs | cFAR: 0.37
mean
[+σ, -σ]
[min, max]
relative frequency
3.45Å
2.60Å
Elevation
Azimuth
# of images
resolution (Å)
Unmasked
Masked
FSC
1/resolution (1/Å)
d
Bridge Helix
2Å
6Å
GSFSC Resolution: 2.88Å
No Mask (3.5Å)
Loose (3.1Å)
Tight (2.9Å)
Corrected (2.9Å)
3072 cFSCs | cFAR: 0.20
3.80Å
2.54Å
Elevation
Azimuth
# of images
resolution (Å)
Unmasked
Masked
FSC
1/resolution (1/Å)

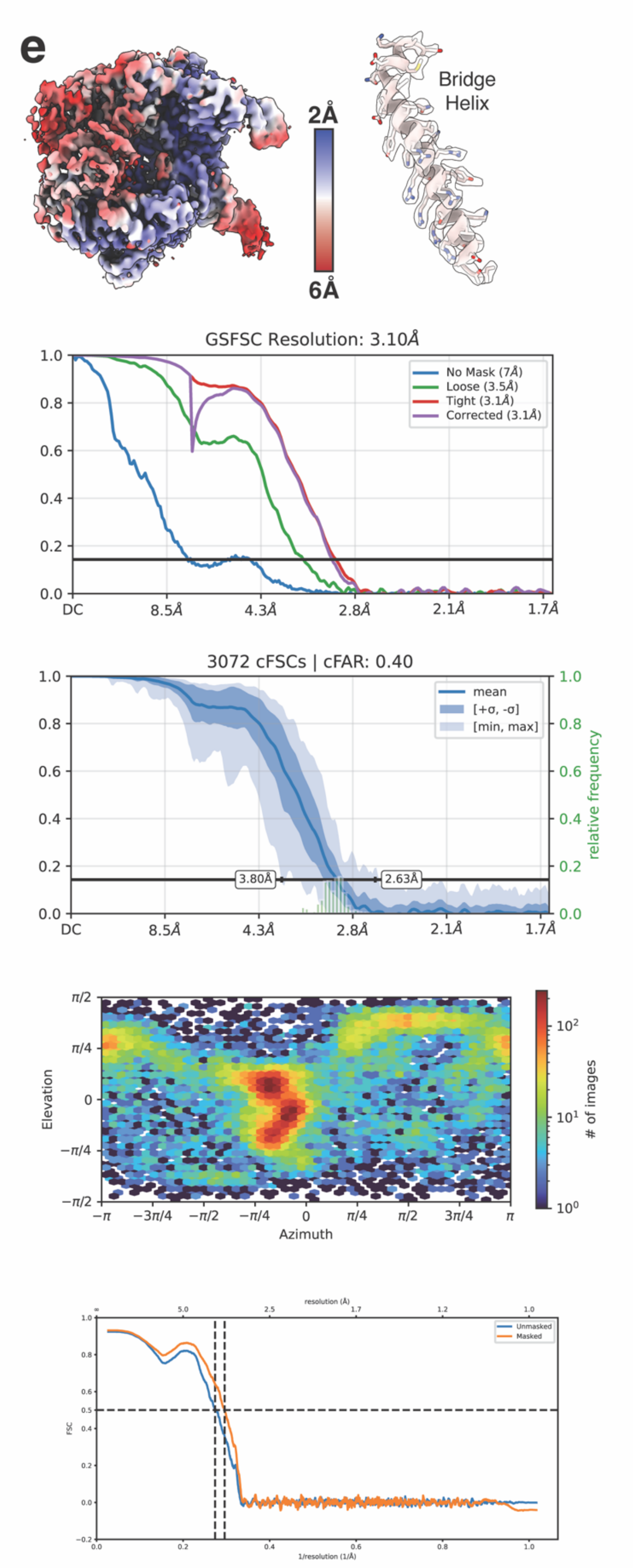

Supplementary Figure 6. Map and model quality assessment. (a-e) From top to bottom: local resolution of cryo-EM reconstructions, representative map quality showing

resolution of bridge helix sidechains, FSC curves, circular FSC curves, angular distribution plots, and map-to-model FSC curves for **(a)** the inactive state, **(b)** the pre-cleavage state, **(c)** the checkpoint state, **(d)** the product state, and **(e)** the dissociated state.

a

rate constant	Best Fit		
	**20-nt**	**15-nt**	**2mm**
k_1	1.0 nM^{-1} min^{-1} *	1.0 nM^{-1} min^{-1} *	1.0 nM^{-1} min^{-1} *
k_{-1}	1.0 min^{-1} *	1.0 min^{-1} *	1.0 min^{-1} *
k_2	[60.0 min^{-1}]	0.109 min^{-1} (0.096, 0.11)	0.723 min^{-1} (0.681, 0.787)
k_{-2}	[0.0001 min^{-1}]	[0.0001 min^{-1}]	[0.0001 min^{-1}]
k_3	0.00085 min^{-1} (0.00077, 0.00092)	0.005 min^{-1} (0.0049, 0.0052)	0.053 min^{-1} (0.049, 0.052)
k_{-3}	{ 1.0 min^{-1} }	{ 0.186 min^{-1} }	{ 1.08 min^{-1} }

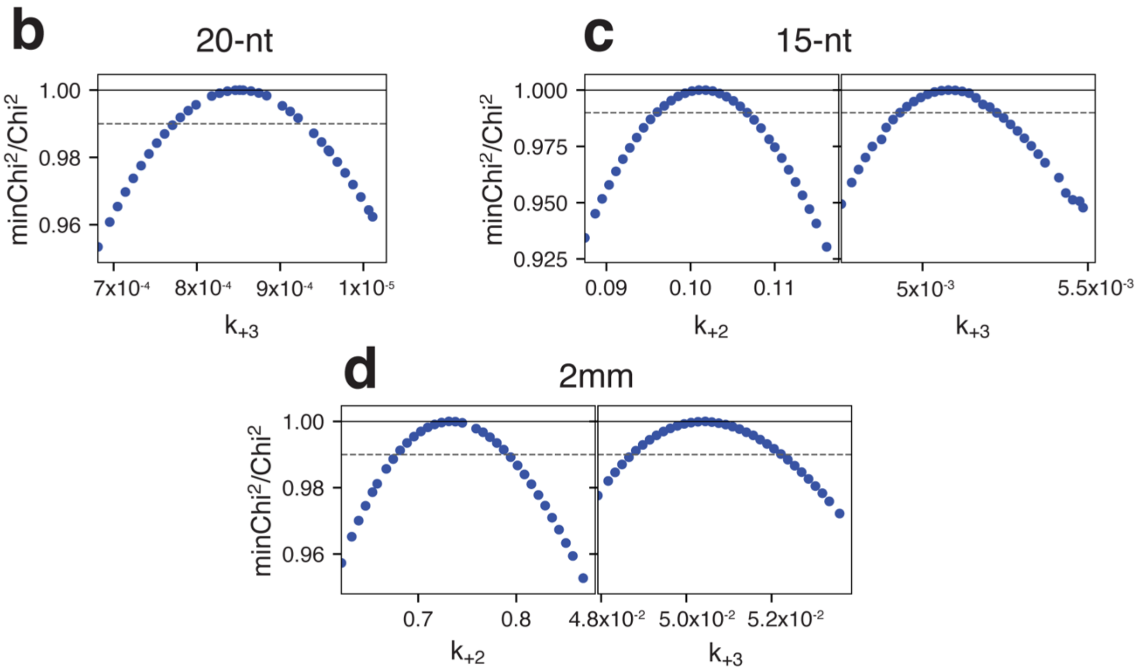

Supplementary Figure 7. (a) Best fit rate constants generated by global fitting. **(b-d)** Confidence contours from global data fitting for **(b)** 15-nt sgRNA sample **(c)** 20-nt sample, and **(d)** 2mm sample. Contours show the change in chi2 as a function of values for individual rate. The dashed line indicates the chi2 threshold corresponding to the 95% confidence interval used for reporting upper and lower limits on parameters listed in panel (a).

	Pre-activation (EMDB-47834) (PDB 9EAK)	Pre-cleavage (EMDB-47835) (PDB 9EAL)	Checkpoint (EMDB-47941) (PDB 9ED9)	Product (EMDB-47942) (PDB 9EDA)	Dissociated (EMDB-47943) (PDB 9EDB)
**Data collection and processing**	81,000	81,000	81,000	81,000	81,000
Magnification	300	300	300	300	300
Voltage (kV)	80	80	80	80	80
Electron exposure (e–/Å^2)	-1.2 to -2.2	-1.2 to -2.2	-1.2 to -2.2	-1.2 to -2.2	-1.2 to -2.2
Defocus range (μm)	0.8332	0.8332	0.8332	0.8332	0.8332
Pixel size (Å)	C_1	C_1	C_1	C_1	C_1
Symmetry imposed	1,564,462	1,564,462	1,564,462	1,564,462	1,564,462
Initial particle images (no.)	112,156	125,923	59,974	180,648	26,205
Final particle images (no.)	2.76	2.83	2.95	2.88	3.10
Map resolution (Å)<br>FSC threshold	0.143	0.143	0.143	0.143	0.143
Map resolution range (Å)	2.5-7	2.5-7	2.5-7	2.5-7	2.5-7
**Refinement**					
Initial model used (PDB code)	6O0Z	7S4V	7Z4L	7S4V	4ZT0
Model resolution (Å)	2.9	3.1	3.1	3.2	3.4
FSC threshold	0.5	0.5	0.5	0.5	0.5
Model resolution range (Å)	N/A	N/A	N/A	N/A	N/A
Map sharpening *B* factor (Å^2)	-61.3	-65.0	-57.1	-65.1	-46.6
Model composition					
Non-hydrogen atoms	12,880	11,498	13,801	12,573	12,644
Protein residues	1,356	1,167	1,337	1,190	1,342
Nucleotides	149	143	135	135	122
Ligands	1	0	0	0	0
B factors (Å^2)					
Protein	113.76	101.31	107.82	105.55	122.04
Nucleotides	129.21	109.23	95.62	108.29	124.34
Ligand	85.04	N/A	N/A	N/A	N/A
R.m.s. deviations					
Bond lengths (Å)	0.004	0.005	0.004	0.006	0.005
Bond angles (°)	0.800	0.874	0.840	1.178	0.895
Validation					
MolProbity score	1.50	1.50	1.42	1.65	1.52
Clashscore	3.74	4.92	4.28	6.85	4.92
Poor rotamers (%)	1.09	0.94	0.58	0.66	0.43
Ramachandran plot					
Favored (%)	95.56	96.34	96.69	96.02	96.09
Allowed (%)	4.44	3.66	3.31	3.98	3.91
Disallowed (%)	0	0	0	0	0

Supplementary Table 1. Cryo-EM data collection, refinement and validation statistics.

Name	Sequence (5'-3')	Source
55bp_TS	AGCTGACGTTTGTACTCCAGCGTCTCATCTTTATGCG TCAGCAGAGATTTCTGCT	IDT
55bp_NTS	AGCAGAAATCTCTGCTGACGCATAAAGATGAGACGCT GGAGTACAAACGTCAGCT	IDT
20nt_sgRNA	GGCGCAUAAAGAUGAGACGCGUUUUAGAGCUAGAA AUAGCAAGUUAAAAUAAGGCUAGUCCGUUAUCAACU UGAAAAAGUGGCACCGAGUCGGUGCUUUU	Genscript
17nt_sgRNA	GCAUAAAGAUGAGACGCGUUUUAGAGCUAGAAAUA GCAAGUUAAAAUAAGGCUAGUCCGUUAUCAACUUG AAAAAGUGGCACCGAGUCGGUGCUUUU	Genscript
16nt_sgRNA	CAUAAAGAUGAGACGCGUUUUAGAGCUAGAAAUAG CAAGUUAAAAUAAGGCUAGUCCGUUAUCAACUUGAA AAAGUGGCACCGAGUCGGUGCUUUU	Genscript
15nt_sgRNA	AUAAAGAUGAGACGCGUUUUAGAGCUAGAAAUAGC AAGUUAAAAUAAGGCUAGUCCGUUAUCAACUUGAAA AAGUGGCACCGAGUCGGUGCUUUU	Genscript
14nt_sgRNA	UAAAGAUGAGACGCGUUUUAGAGCUAGAAAUAGCA AGUUAAAAUAAGGCUAGUCCGUUAUCAACUUGAAAA AGUGGCACCGAGUCGGUGCUUUU	Genscript
15mm1_sgRNA	GAUAAAGAUGAGACGCGUUUUAGAGCUAGAAAUAG CAAGUUAAAAUAAGGCUAGUCCGUUAUCAACUUGAA AAAGUGGCACCGAGUCGGUGCUUUU	Genscript
15mm2_sgRNA	CGAUAAAGAUGAGACGCGUUUUAGAGCUAGAAAUA GCAAGUUAAAAUAAGGCUAGUCCGUUAUCAACUUG AAAAAGUGGCACCGAGUCGGUGCUUUU	Genscript

Supplementary Table 2. DNA and sgRNA sequences used in this study.

Supplementary Source Data

Uncropped gels from **(a)** Figure 4f **(b)** Supplementary Figure 1a-c, and **(c)** Supplementary Figure 1h. Boxes indicate regions that were cropped and shown in the figures.

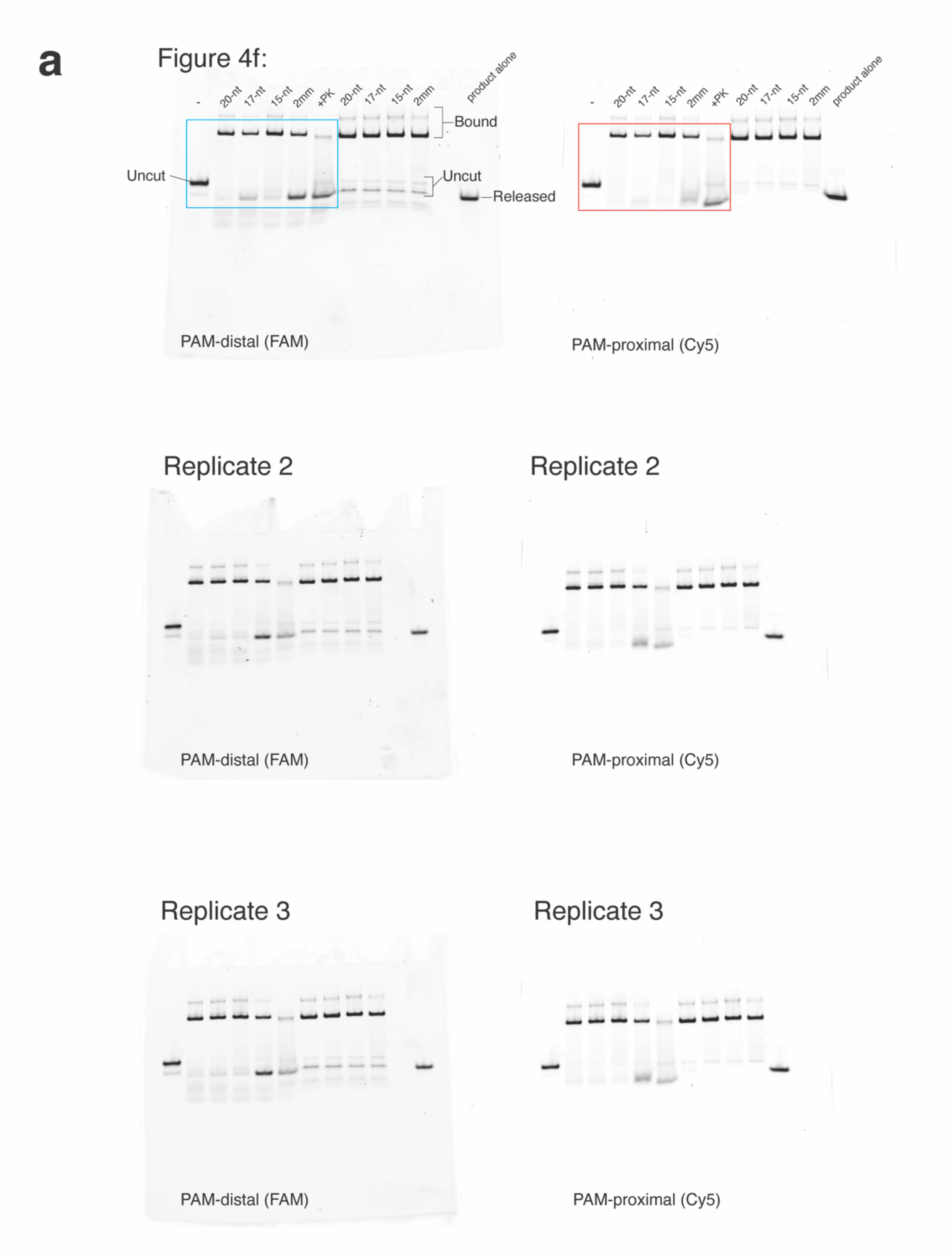

b Supplementary Figure 1a:

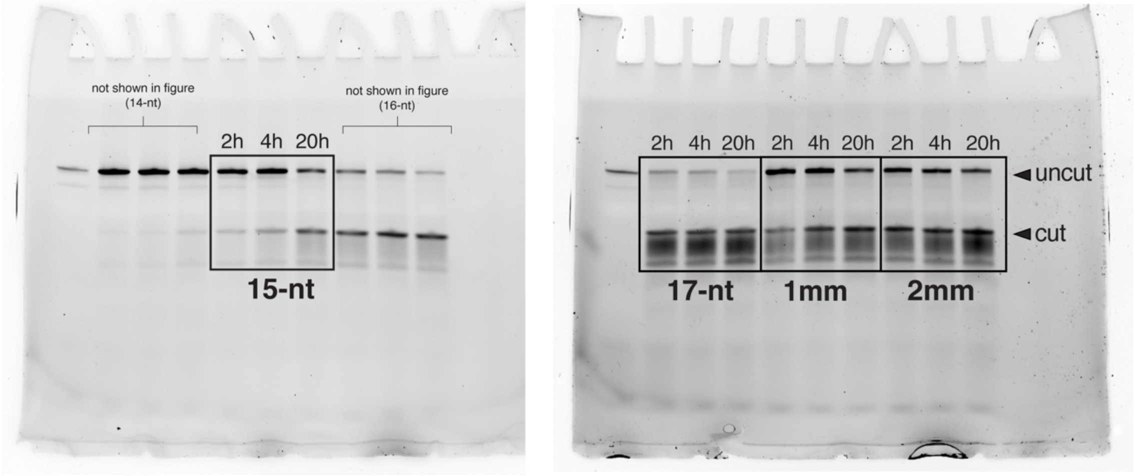

Supplementary Figure 1b:

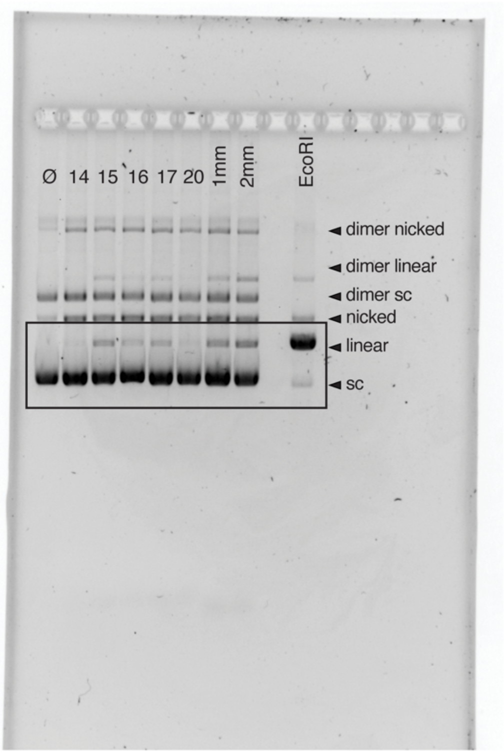

Supplementary Figure 1d:

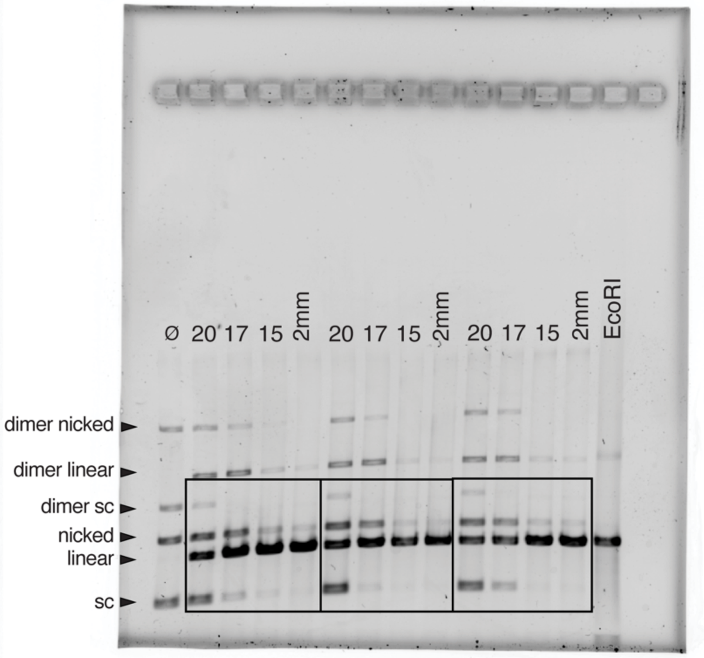

c Supplementary Figure 1h:

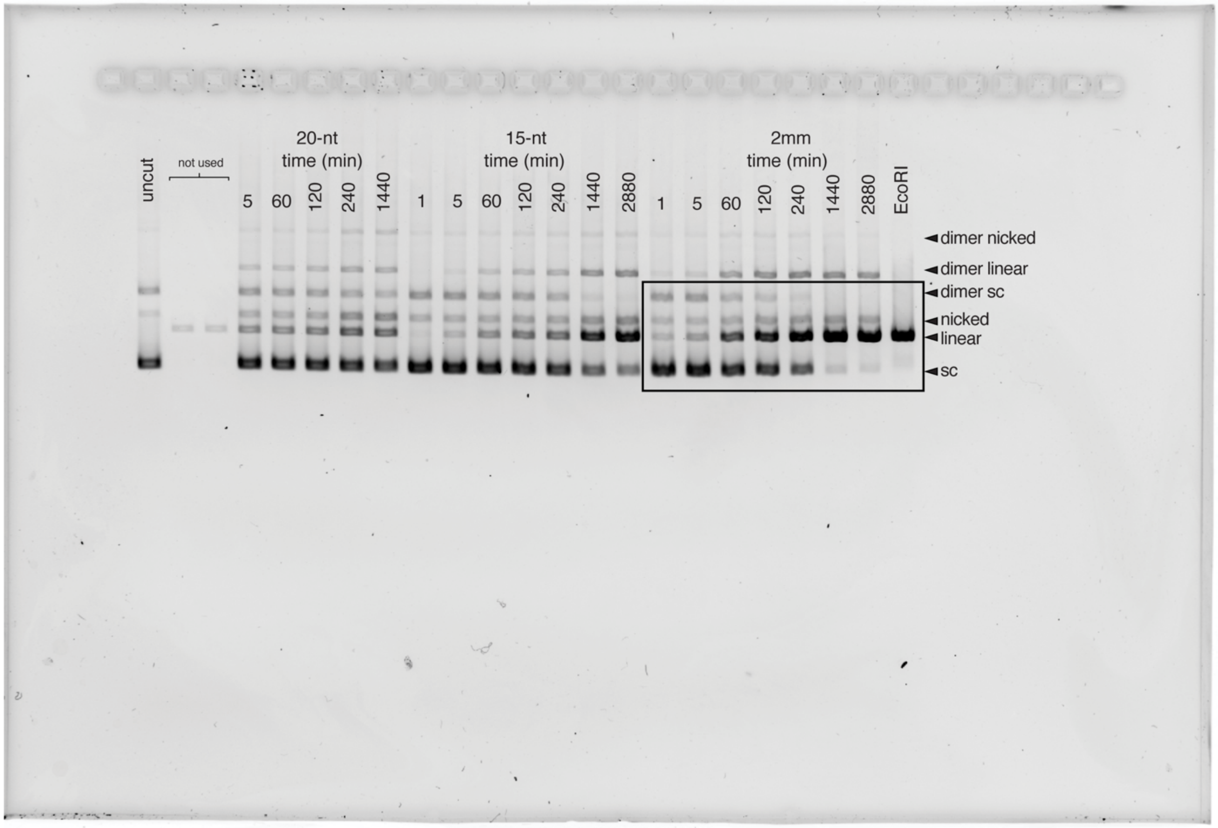

Supplement: Supplementary file 1 — Supplementary Information [file 41467_2025_60668_MOESM1_ESM.pdf]
